# ESRP1 is overexpressed in ovarian cancer and promotes switching from mesenchymal to epithelial phenotype in ovarian cancer cells

**DOI:** 10.1038/oncsis.2017.87

**Published:** 2017-10-09

**Authors:** H M Jeong, J Han, S H Lee, H-J Park, H J Lee, J-S Choi, Y M Lee, Y-L Choi, Y K Shin, M J Kwon

**Affiliations:** 1 Laboratory of Molecular Pathology and Cancer Genomics, College of Pharmacy, Seoul National University, Seoul, Korea; 2 Gencurix Inc., 242 Digital-ro, Seoul, Korea; 3 Research Institute of Pharmaceutical Sciences, College of Pharmacy, Kyungpook National University, Daegu, Korea; 4 The Center for Anti-cancer Companion Diagnostics, Bio-MAX/N-Bio, Seoul National University, Seoul, Korea; 5 BK21 Plus KNU Multi-Omics based Creative Drug Research Team, Research Institute of Pharmaceutical Sciences, College of Pharmacy, Kyungpook National University, Daegu, Korea; 6 Laboratory of Cancer Genomics and Molecular Pathology, Samsung Medical Center, Sungkyunkwan University School of Medicine, Seoul, Korea; 7 Department of Pathology and Translational Genomics, Samsung Medical Center, Sungkyunkwan University School of Medicine, Seoul, Korea; 8 Department of Health Sciences and Technology, SAIHST, Sungkyunkwan University, Seoul, Korea; 9 Department of Molecular Medicine and Biopharmaceutical Sciences, Graduate School of Convergence Science and Technology, Seoul National University, Seoul, Korea; 10 College of Pharmacy, Kyungpook National University, Daegu, Korea

## Abstract

Epithelial splicing regulatory protein 1 (ESRP1) and 2 (ESRP2), epithelial cell-specific regulators of alternative splicing, are downregulated during the epithelial–mesenchymal transition (EMT). These factors have roles in tumor progression and metastasis in some cancers; however, their expression and function in ovarian cancer (OC) remain unclear. We found that *ESRP1* and *ESRP2* mRNAs were expressed at higher levels in OC cells than in immortalized ovarian surface epithelial (IOSE) cells, and confirmed their overexpression in OC tissues at the protein level. The Cancer Genome Atlas (TCGA) data analysis revealed frequent gene amplification of *ESRP1* in OC tissues; however, we detected no significant correlation between *ESRP1* gene copy number and gene expression in OC cells. Importantly, expression of *ESRP1* and *ESRP2* was inversely correlated with DNA methylation in OC cells, and ESRP2 overexpression in OC tissues was significantly associated with DNA hypomethylation. Notably, survival analysis using TCGA data from 541 OC tissues revealed that high *ESRP1* expression was significantly associated with shorter 5-year survival of patients. Ectopic ESRP1 expression in mesenchymal OC cells promoted cell proliferation but suppressed cell migration. Furthermore, we found that ESRP1 drives a switch from mesenchymal to epithelial phenotype characterized by reduced cell migration in association with induction of epithelial cell-specific variant of *CD44* and *ENAH*. Taken together, our findings suggest that an epigenetic mechanism is involved in ESRP1 overexpression, and that ESRP1 has a role in OC progression.

## Introduction

Epithelial splicing regulatory proteins (ESRP1 and ESRP2) are epithelial cell-specific RNA-binding proteins that regulate alternative splicing of multiple genes, including *CD44*, *CTNND1, ENAH* and *FGFR2*, and thus participate in the epithelial–mesenchymal transition (EMT).^1, 2^ The EMT, which augments tumor motility and invasiveness, has a critical role in metastasis by facilitating the escape of cancer cells from primary tumors.^3, 4, 5^ Moreover, accumulating evidence indicates that the EMT promotes the emergence of cancer stem cells or tumor-initiating cells, implying that this process contributes to drug resistance and recurrence in human cancer.^6, 7^

Expression of ESRP1 and ESRP2 is downregulated during the EMT, and repression of these genes is mediated by EMT-related transcription factors such as SNAI1, SNAI2, ZEB1, ZEB2 and TWIST. Previous studies revealed that SNAI1 and ZEB1 repress ESRP1 at a transcriptional level,^8, 9^ whereas ESRP2 is repressed by ZEB1 or ZEB2.^10^ Recently, other regulatory mechanisms were also discovered. In normal and lung cancer cells, oncogenic EMLK-ALK kinase regulates ESRP1 and ESRP2 expression.^11^ Arkadia, a RING-type E3 ligase, is involved in the ubiquitination of ESRP2,^12^ and in this manner regulates ESRP2 expression at the post-transcriptional level in clear-cell renal cell carcinoma cells.

Consistent with the involvement of ESRP1 and ESRP2 in the EMT, recent studies have revealed the roles and clinical significance of these factors in tumor progression and metastasis. They have been frequently reported as tumor suppressors in various cancers. In human head and neck carcinoma cells, ESRP1 and ESRP2 decrease cell motility,^13^ and ESRP2 inhibits the proliferation of clear-cell renal cell carcinoma cells.^12^ In agreement with these *in vitro* findings, ESRP1 suppresses tumorigenic potential in colorectal cancer^14^ and attenuates liver metastases in pancreatic cancer *in vivo*.^15^ Moreover, ESRP1 expression is a favorable prognostic factor in pancreatic cancer.^15^ In colorectal cancer, both ESRP1 and ESRP2 expression correlate with favorable outcome.^16^ Survival analysis using The Cancer Genome Atlas (TCGA) RNA-sequencing data revealed a significant association between high *ESRP1* gene expression and longer patient survival in clear-cell renal cell carcinoma and breast cancer.^17^ Interestingly, another recent study analyzing TCGA RNA-sequencing data showed that the expression of some ESRP2-targeted exons correlates with favorable prognosis, whereas *ESRP2* expression is not associated with overall survival (OS) rate of clear-cell renal cell carcinoma patients.^12^

However, pro-oncogenic role of ESRP1 has also been reported. ESRP1 promotes lung metastasis by regulating the alternative splicing of *CD44* mRNA, and high *ESRP1* gene expression correlates with significantly shorter OS in breast cancer patients.^18^ ESRP1-low melanomas are associated with favorable patient survival.^19^ Low ESRP1 expression in melanoma also correlates with elevated immune cytotoxicity, suggesting that ESRP1 could serve as a biomarker for immunotherapy as well as a prognostic marker.^19^ Moreover, in contrast to previous studies,^14, 16^ Fagoonee *et al.*^20^ demonstrated that ESRP1 overexpression promotes colorectal cancer progression by stimulating growth of cancer cell. These studies indicated that ESRP1 or ESRP2 may have opposing roles in different tumor types, but their roles and clinical significance in ovarian cancer (OC) remains to be elucidated.

In this study, using TCGA data, we first analyzed the expression of *ESRP1* and *ESRP2* in OC tissues in comparison with normal ovaries, and validated their expression at the protein level in OC cells and tissues. We then examined the molecular mechanism underlying upregulation of *ESRP1* or *ESRP2* in OC, using gene copy number and DNA methylation analysis. We also investigated the association of *ESRP1* expression with clinical outcome using TCGA data and further characterized the role of ESRP1 in OC cells.

## Results

### ESRP1 and ESRP2 are upregulated in human OC cell lines and tissues

**Figure 1:**
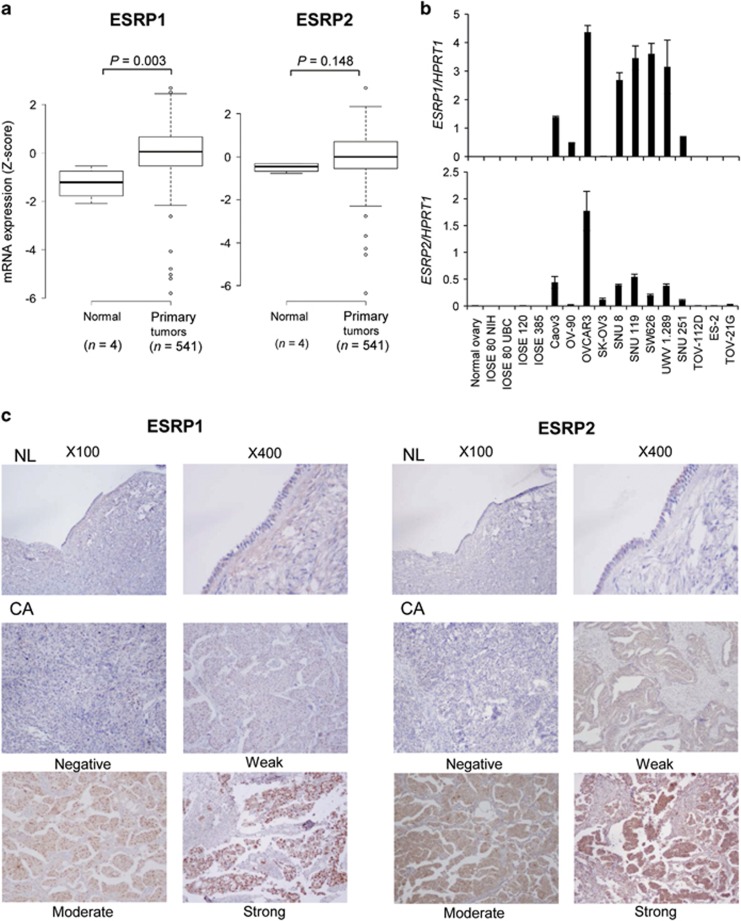
*ESRP1* and *ESRP2* gene expression in human ovarian cancer cell lines, and protein expression in ovarian serous adenocarcinoma. (**a**) Box plot comparing the gene expression of *ESRP1* and *ESRP2* between normal and ovarian cancer tissues using TCGA data. The horizontal line within the box indicates the median, boundaries of the box indicate the 25th and 75th percentile and the whiskers indicate the highest and lowest values of the results. Statistical differences between the two groups were evaluated using the Mann–Whitney test. (**b**) *ESRP1* and *ESRP2* gene expression in ovarian cell lines, determined by qRT–PCR. Data are presented as the mean±s.d. of two or three experiments. (**c**) Representative immunohistochemical staining of human ovarian tissues with anti-ESRP1 or anti-ESRP2 antibodies. ESRP1 (left) and ESRP2 (right) in normal ovarian surface epithelium and ovarian serous adenocarcinoma tissues. Magnification, × 100 or × 400. CA, carcinoma; NL, normal.

We first analyzed the gene expression of *ESRP1* and *ESRP2* in OC tissues in comparison with normal ovaries using TCGA data based on Agilent gene expression microarrays. TCGA data revealed that *ESRP1* gene expression is significantly higher in primary ovarian serous cystadenocarcinoma (*n*=541) than normal ovarian tissues (*n*=4; *P*=0.003; Figure 1a). In the case of *ESRP2*, we detected a trend toward higher expression in OC, but it was not significant. On the basis of the TCGA data, we determined the transcript levels of *ESRP1* and *ESRP2* in OC cell lines using real-time quantitative reverse transcription–PCR (qRT–PCR). qRT–PCR data confirmed that *ESRP1* and *ESRP2* mRNA levels were upregulated in OC cell lines in comparison with normal ovaries and immortalized ovarian surface epithelial (IOSE) cells (Figure 1b).

To further validate the expression of ESRP1 and ESRP2 at the protein level, we performed immunohistochemical analysis in formalin-fixed, paraffin-embedded (FFPE) OC tissues. ESRP1 and ESRP2 were weakly expressed in normal ovarian surface epithelium, and their levels were frequently elevated in OC tissues (Figure 1c). ESRP1 expression was mainly detected in the nucleus of OC cells. Of the 69 cases of ovarian serous adenocarcinomas examined for ESRP1, 53 (76.8%) cases showed moderate or strong expression with higher expression than normal ovaries (Figure 1c, left). In the case of ESRP2, expression was mainly observed in the cytoplasm rather than the nucleus, and 36 of the 59 cases (61.0%) showed moderate expression in the cytoplasm of OC cells (Figure 1c, right). These results confirmed that both ESRP1 and ESRP2 are overexpressed in OC tissues.

### *ESRP1* gene copy number in OC cell lines and tissues

**Figure 2:**
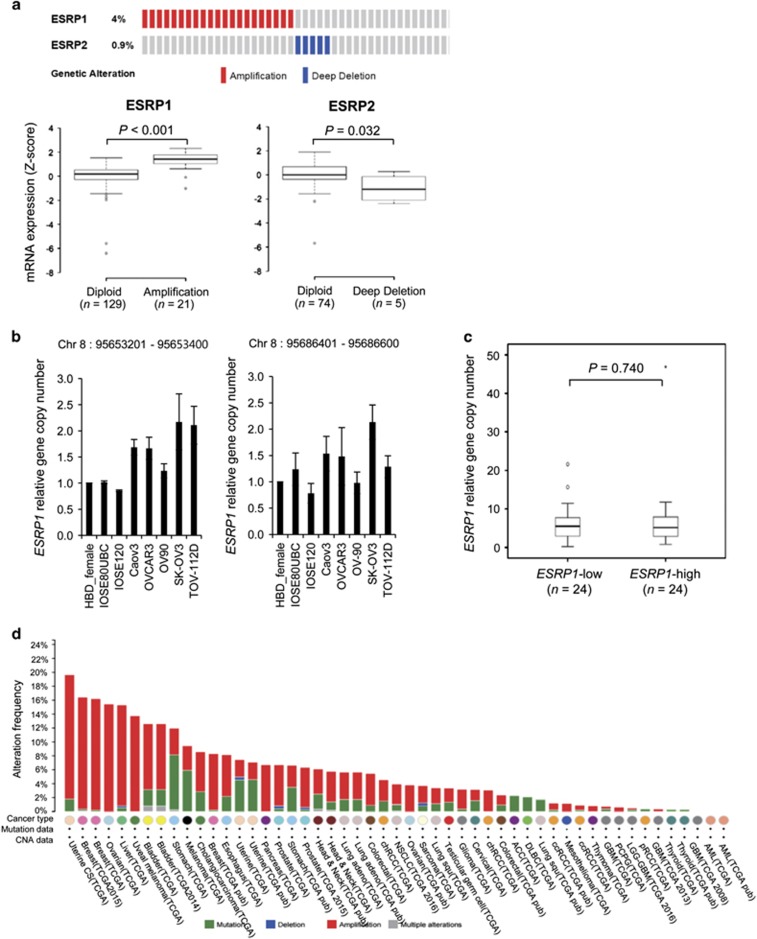
Genetic alterations of *ESRP1* or *ESRP2* in ovarian cancer tissues. (**a**) OncoPrint of gene copy number alterations of *ESRP1* or *ESRP2* in 557 ovarian cancer samples from TCGA data^23^ (top), and box plot showing the association between their mRNA levels and gene amplification or deletion (bottom). OncoPrint was generated using cBioPortal (http://www.cbioportal.org/). Statistical differences between the two groups were evaluated using the Mann–Whitney test. (**b**) *ESRP1* gene copy number analysis in ovarian cancer cells. Gene copy numbers for two regions (Chr8 95653201–95653400 and 95686401–95686600, GRCh37) within the genomic region encoding *ESRP1* were determined using qPCR. Data are presented as the mean±s.d. of two experiments. HBD_female, Human female blood gDNA. (**c**) Box plot comparing the *ESRP1* gene copy number for region (Chr8 95653201–95653400) between the *ESRP1-*low and -high groups of ovarian cancer FFPE tissues. Statistical differences between the two groups were evaluated using the Mann–Whitney test. (**d**) *ESRP1* genetic alterations in various cancer types using cBioPortal based on TCGA data. ACC, adrenocortical carcinoma; AML, acute myeloid leukemia; chRCC, chromophobe renal cell carcinoma; DLBC, diffuse large B-cell lymphoma; GBM, glioblastoma multiforme; LGG-GBM, lower-grade glioma-GBM; NSCLC, non-small cell lung cancer; PCPG, pheochromocytoma and paraganglioma; pRCC, papillary renal cell carcinoma; Uterine CS, uterine carcinosarcoma.

Next, we sought to elucidate the molecular mechanism underlying *ESRP1* and *ESRP2* overexpression in OC. Genetic alterations of *ESRP1* and *ESRP2* were analyzed using cBioPortal^21, 22^ based on TCGA data for OC used in a previous study.^23^ Copy number alteration data revealed *ESRP1* gene amplification (4%, 21/557) and *ESRP2* deletion (0.9%, 5/557) in 557 OC tissues (Figure 2a, top). Combined analysis of copy number alteration and gene expression data revealed that *ESRP1* mRNA level is significantly higher in *ESRP1*-amplified OC tissues than in non *ESRP1*-amplified OC tissues (*P*<0.001), whereas *ESRP2* deletion is significantly associated with lower *ESRP2* gene expression (Figure 2a, bottom). These results suggested that gene amplification may be a mechanism involved in ESRP1 overexpression. Accordingly, we examined the copy number of *ESRP1* in IOSE cells and OC cell lines using qPCR. Two regions within the genomic region encoding *ESRP1* were used for gene copy number analysis. Gene copy numbers of *ESRP1* were higher in OC cell lines than in IOSE cells (Figure 2b). However, we observed no significant correlation between *ESRP1* expression and gene copy number in OC cell lines or FFPE tissues (Figures 2b and c). We then analyzed the frequency of genetic alteration of *ESRP1* in various human cancers, and found that *ESRP1* gene amplification was common in female cancers including uterine carcinosarcoma, breast and OC (Figure 2d).

### DNA hypomethylation is associated with ESRP1 or ESRP2 overexpression in OC cells

Our genetic alteration analysis using TCGA data and *ESRP1* gene copy number assays indicated that gene amplification did not explain the overexpression of *ESRP1* or *ESRP2* in OC. Hence, we performed genome-wide DNA methylation analysis using Illumina HumanMethylation450 BeadChips (Illumina, San Diego, CA, USA). We detected DNA hypomethylation of CpG sites in the promoter region in OC cells expressing high levels of *ESRP1* or *ESRP2* (hereafter, *ESPR1*- or *ESPR2*-high), but DNA hypermethylation in cells expressing low levels of *ESRP1* or *ESRP2* (hereafter, *ESPR1*- or *ESPR2*-low), relative to the DNA methylation levels in two IOSE cell lines (data not shown). On the basis of our DNA methylation microarray data, we investigated whether epigenetic mechanisms are involved in *ESRP1* or *ESRP2* overexpression in OC.

**Figure 3:**
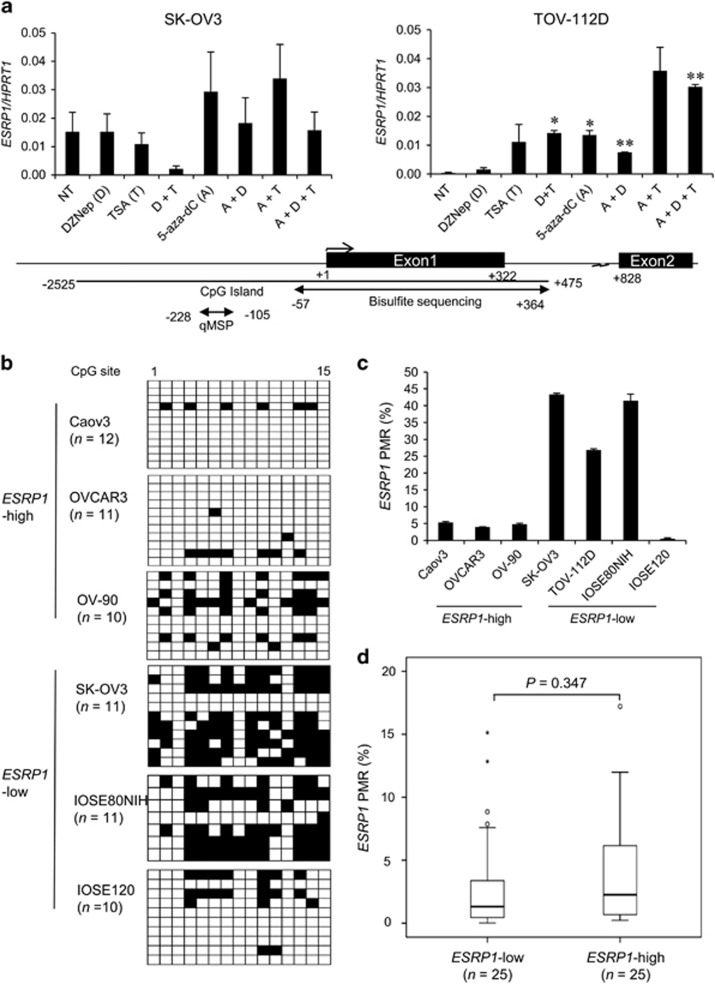
DNA methylation status in the promoter region of *ESRP1* in ovarian cancer cell lines and tissues. (**a**) *ESRP1* gene expression in ovarian cancer cells following treatment with epigenetic drugs. Data for qRT–PCR are presented as the mean±s.d. of two or three experiments. *ESRP1* expression following treatment with epigenetic drugs was compared with that with no treatment (NT). Student’s *t*-test; *P<0.05, **P<0.01. (**b**) DNA methylation analysis of IOSE and ovarian cancer cells using bisulfite sequencing and (**c**) qMSP. DNA methylation level is expressed as percentage of methylated reference (PMR, %) values. Data for qMSP are presented as the mean±s.d. of two experiments. (**d**) Box plot showing the association between DNA methylation level (PMR) and *ESRP1* expression in ovarian cancer FFPE tissues. Statistical differences between the two groups were evaluated using the Mann–Whitney test.

First, we treated *ESRP1-* or *ESRP2-*low cells with epigenetic drugs, specifically, a histone methyltransferase inhibitor (3-deazaneplanocin A), DNA methyltransferase inhibitor (5-aza-2′-deoxycytidine) and a histone deacetylase inhibitor (trichostatin A). *ESRP1* transcript levels were significantly elevated by 5-aza-2′-deoxycytidine treatment alone in TOV-112D cells (Figure 3a). To confirm DNA methylation status in the *ESRP1* promoter region, we performed bisulfite sequencing and quantitative methylation-specific PCR (qMSP). Bisulfite sequencing of a total of 15 CpG sites across 421 bp within CpG islands of the *ESRP1* promoter region revealed DNA hypermethylation in *ESRP1*-low IOSE cells and OC cells, but a low level of DNA methylation in *ESRP1*-high OC cells (Figures 3b and c). These results revealed the inverse correlation between *ESRP1* expression and DNA methylation in OC cells. On the basis of this correlation, we further analyzed *ESRP1* DNA methylation in OC FFPE tissues (*n*=50), but we detected no significant difference (Figure 3d).

**Figure 4:**
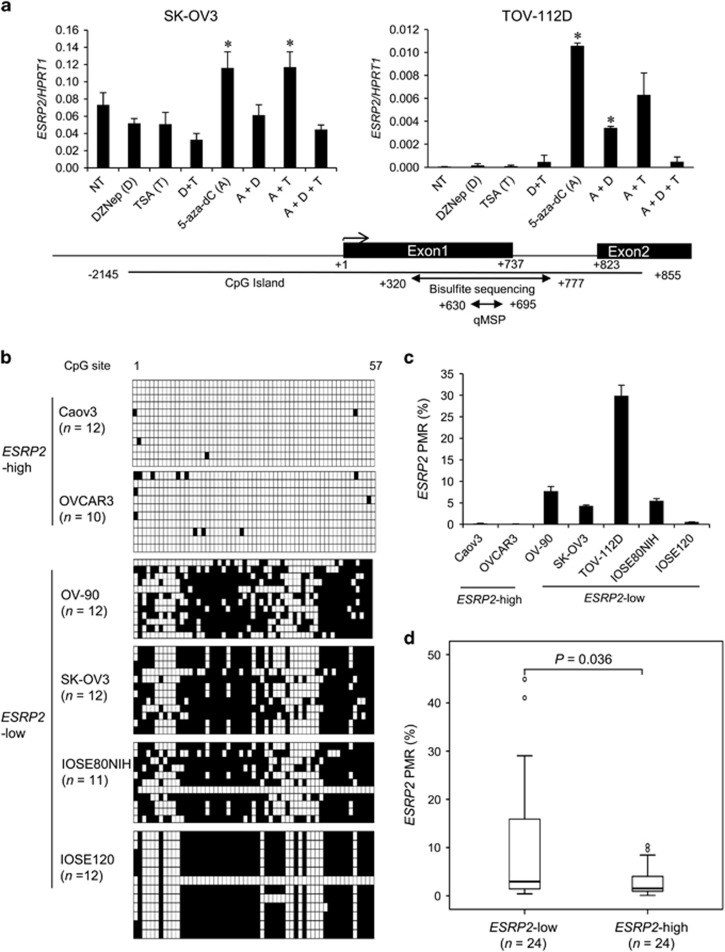
DNA methylation status in the promoter region of *ESRP2* in ovarian cancer cell lines and tissues. (**a**) *ESRP2* gene expression following treatment with epigenetic drugs. Data for qRT–PCR are presented as the mean±s.d. of two or three experiments. *ESRP2* expression following treatment with epigenetic drugs was compared with that with no treatment (NT). Student’s *t*-test, *P<0.05. (**b**) DNA methylation analysis of IOSE and ovarian cancer cells using bisulfite sequencing and (**c**) qMSP. Data for qMSP are presented as the mean±s.d. of two experiments. (**d**) Box plot showing the association between DNA methylation and *ESRP2* gene expression in ovarian cancer FFPE tissues. Statistical differences between the two groups were evaluated using the Mann–Whitney test.

Similar to *ESRP1*, *ESRP2* transcript levels were significantly elevated in *ESRP2*-low OC cells upon treatment with DNA methyltransferase inhibitor alone (Figure 4a). Bisulfite sequencing in 57 CpG sites across 458 bp within CpG islands in the promoter region of *ESRP*2 and qMSP results also confirmed higher DNA methylation levels in *ESRP2-*low cells than in *ESRP2*-high cells (Figures 4b and c). Importantly, the *ESRP2-*high group (*n*=25) of OC FFPE tissues had a significantly lower DNA methylation level than the *ESRP1-*low group (*n*=25; *P*=0.036; Figure 4d). Taken together, these findings demonstrate that DNA hypomethylation correlates with both high *ESRP1* and *ESRP2* expression in OC cells, but is significantly associated with high *ESRP2* mRNA level, but not *ESRP1* mRNA level, in OC FFPE tissues.

### Higher *ESRP1* gene expression is associated with shorter patient survival in OC

**Figure 5:**
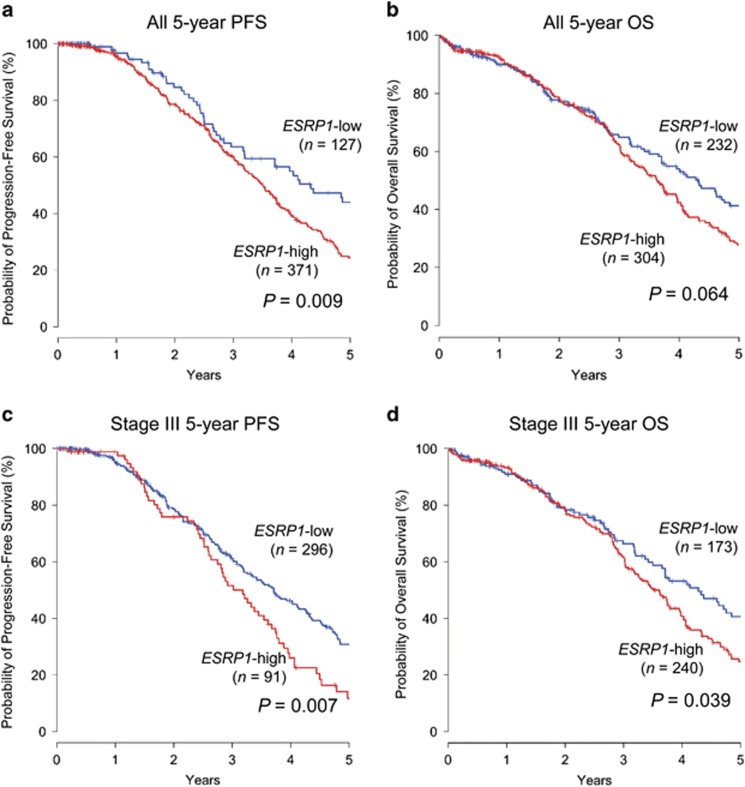
Association between *ESRP1* gene expression and OS or PFS in patients with ovarian serous adenocarcinoma. (**a**) Kaplan–Meier plot of 5-year OS, and PFS in total primary tumors and (**b**) stage III primary tumors. (**a, b**) Kaplan–Meier plot of 5-year OS, and PFS in total primary tumors and (**c, d**) stage III primary tumors.

On the basis of our TCGA data analysis revealing *ESRP1* amplification in OC tissues, we hypothesized that ESRP1 has a pro-oncogenic role in OC. Accordingly, using TCGA data, we analyzed the association of *ESRP1* expression with clinical outcome in patients with ovarian serous cystadenocarcinoma (*n*=541). The median age of patients was 59.6 years (26.0–89.0). Of 541 cases, the majority of patients (76.7%, 415/541) had stage III tumors, followed by 14.4% (78/541) with stage IV tumors. Histologically, the majority of tumors (83.4%, 451/541) were grade 3 tumors. The median follow-up duration was 3.75 years (3.50–4.07) for OS and 3.64 years (3.32–3.96) for progression-free survival (PFS), respectively. Patients were divided into *ESRP1*-high and *ESRP1*-low groups, and survival analysis was performed using the Kaplan–Meier method. The *ESRP1*-high group had a significantly shorter 5-year PFS than the ESRP1-low group (log-rank test, *P*=0.009; Figure 5a). A marginally significant difference between groups was observed for 5-year OS (*P*=0.064; Figure 5b). Subgroup analysis according to pathologic stage revealed that high *ESRP1* expression was significantly associated with shorter 5-year PFS (*P*=0.007) and OS (*P*=0.039) in patients with stage III OC (Figures 5c and d).

### ESRP1 promotes cell proliferation and suppresses cell migration in OC cells

**Figure 6:**
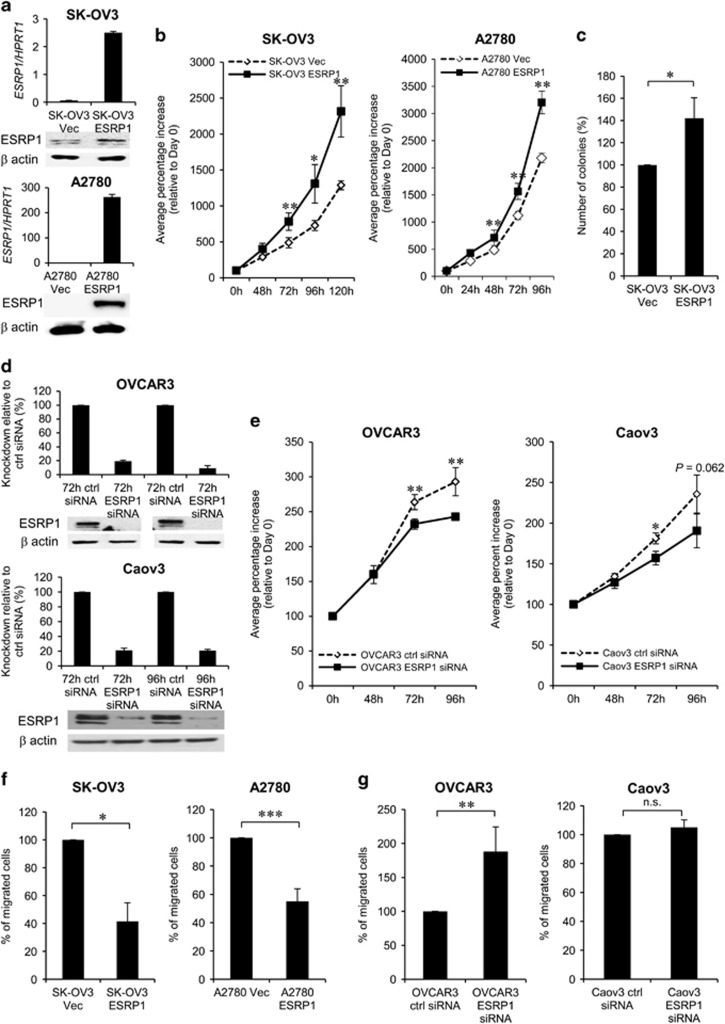
Effect of ESRP1 on cell proliferation and migration in ovarian cancer cells. (**a**, **b**) Effect of enforced ESRP1 expression on cell proliferation in SK-OV3 and A2780 cells. (**c**) Soft agar formation assay in SK-OV3 stable cell lines. (**d**, **e**) Effect of ESRP1 knockdown on cell proliferation in OVCAR3 and Caov3 cells. ESRP1 overexpression or ESRP1 knockdown by siRNA treatment was determined using qRT–PCR and western blot. For *in vitro* cell proliferation assay, viable cell numbers were counted at each time point. (**f**) Effect of enforced ESRP1 expression or (**g**) ESRP1 knockdown on cell migration. Data for cell proliferation and migration are presented as the mean±s.d. of three or four experiments. Student’s *t*-test, *P<0.05, **P<0.01, ***P<0.001, n.s., not significant.

To understand the association between high *ESRP1* expression and shorter survival of patients with OC, we investigated the role of ectopic ESRP1 expression in OC cells. For this purpose, we transfected ESRP1 into SK-OV3 cells, which have low endogenous ESRP1 levels, and established stable cell lines including ESRP1-overexpressing clones (SK-OV3-ESRP1) and clones harboring the empty vector (SK-OV3-Vec). ESRP1 overexpression was confirmed by both qRT–PCR and western blot analysis (Figure 6a).

Using these cell lines, we first tested whether enforced ESRP1 expression affects cell proliferation. SK-OV3-ESRP1 cells exhibited significantly accelerated cell proliferation in comparison with SK-OV3-Vec cells (Figure 6b). To further determine whether ESRP1 overexpression results in anchorage-independent growth, soft agar formation assay was performed. The results showed the significant increase in the number of colonies in SK-OV3-ESRP1 cells compared with SK-OV3-Vec cells (*P*=0.019; Figure 6c). The effect of ESRP1 overexpression on cell proliferation was also tested in A2780 cells with low ESRP1 expression. Transient ESRP1-transfected A2780 cells exhibited significantly increased cell proliferation (Figure 6b). Next, we evaluated the effect of ESRP1 knockdown by small interfering RNA (siRNA) treatment on cell proliferation in ESRP1-high OVCAR3 and Caov3 cells. Significant knockdown of ESRP1 expression by ESRP1 siRNA was also confirmed in two OC cells (Figure 6d). ESRP1 knockdown in OVCAR3 or Caov3 cells significantly inhibited cell proliferation (Figure 6e).

We also investigated the effect of ESRP1 expression on cell migration. SK-OV3-ESRP1 cells exhibited significantly less migration than SK-OV3-Vec cells (*P*=0.017; Figure 6f). ESRP1 overexpression in A2780 cells also significantly inhibited cell migration (*P*<0.001). Conversely, *ESRP1* knockdown in OVCAR3 cells significantly promoted cell migration (*P*=0.003; Figure 6g). Cell migration was not affected by ESRP1 knockdown in Caov3 cells (Figure 6g). These data indicated that enforced ESRP1 expression promotes cell proliferation, but suppresses cell migration, in OC cells.

### ESRP1 drives a switch from mesenchymal to epithelial phenotype in association with upregulation of *CDH1* expression and alternative splicing of *ENAH*

**Figure 7:**
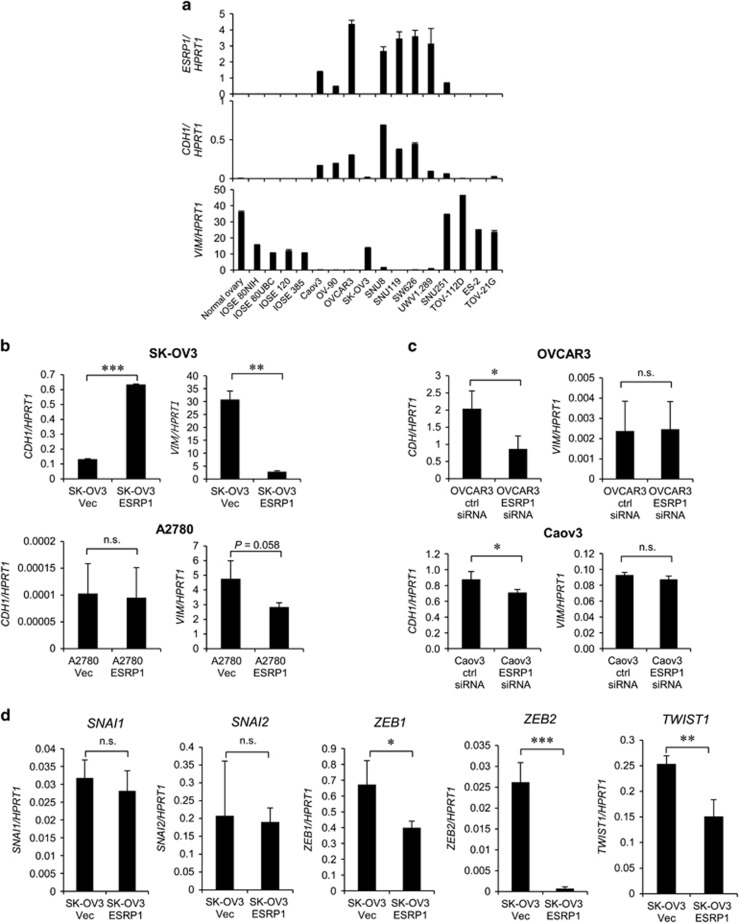
Regulation of EMT markers and EMT-associated transcription factors by ectopic ESRP1 expression. (**a**) Association between *ESRP1* and EMT markers' (*CDH* and *VIM*) gene expression in ovarian cell lines. Data are presented as the mean±s.d. of two experiments. (**b**) *CDH1* or *VIM* gene expression upon ectopic ESRP1 expression in SK-OV3 and A2780 cells or (**c**) ESRP1 knockdown in OVCAR3 and Caov3 cells. (**d**) The mRNA levels of *SNAI1, SNAI2, ZEB1, ZEB2* and *TWIST1* in SK-OV3-Vec and SK-OV3-ESRP1 cells. qRT–PCR data (except for Figure 7a) are presented as the mean±s.d. of three or four experiments. Student’s *t*-test, *P<0.05, **P<0.01, ***P<0.001, n.s., not significant.

We next investigated the mechanism by which ESRP1 inhibits cell migration and promotes cell proliferation. Downregulation of ESRP1 is involved in the EMT, which is characterized by changes in cell morphology, loss of cell–cell interaction and polarity, and elevated cell motility. Therefore, EMT-associated markers may also mediate the reduction in cell migration induced by ESRP1. To test this idea, we examined the relationship between *ESRP1* expression with EMT markers such as *CDH1* and *VIM* in OC cells. Expression of *ESRP1* positively correlated with expression of *CDH1*, but was inversely associated with *VIM* expression (Figure 7a). On the basis of this association, we investigated whether the expression of EMT markers is regulated by ESRP1. *CDH1* was upregulated 4.8-fold in SK-OV3-ESRP1 cells in comparison with SK-OV3-Vec cells, whereas *VIM* expression was substantially downregulated (10.9-fold) in SK-OV3-ESRP1 cells (Figure 7b). Reduced *VIM* expression was also observed in transient ESRP1-transfected A2780 cells. Conversely, *CDH1* expression was downregulated in ESRP1-knockdown OVCAR3 and Caov3 cells (Figure 7c). However, endogenous *CDH1* expression was very low in A2780 cells and was not affected by enforced ESRP1 expression. ESRP1 knockdown in OVCAR3 or Caov3 cells caused no change in the *VIM* mRNA level.

To further elucidate the mechanism underlying upregulation of *CDH1* by ESRP1 in OC cells, we assessed the mRNA levels of EMT-associated zinc-finger transcription factors known to repress *CDH1* expression. The transcript levels of *ZEB1*, *ZEB2* and *TWIST1* were significantly lower in SK-OV3-ESRP1 cells (Figure 7d), whereas no significant difference was observed for *SNAI1* and *SNAI2* expression. In particular, *ZEB2* mRNA levels were substantially downregulated (36.8-fold) in SK-OV3-ESRP1 cells, whereas in *ZEB1* (1.7-fold) and *TWIST1* (1.7-fold) were minimally downregulated. This may indicate that, among the EMT-associated transcription factors, ZEB2 is the most relevant one to the upregulation of *CDH1* expression upon ESRP1 overexpression. Therefore, our results show that the reduced cell migration resulting from enforced ESRP1 expression in OC cells is mediated by *CDH1* upregulation via suppression of EMT-associated transcription factors, especially ZEB2.

**Figure 8:**
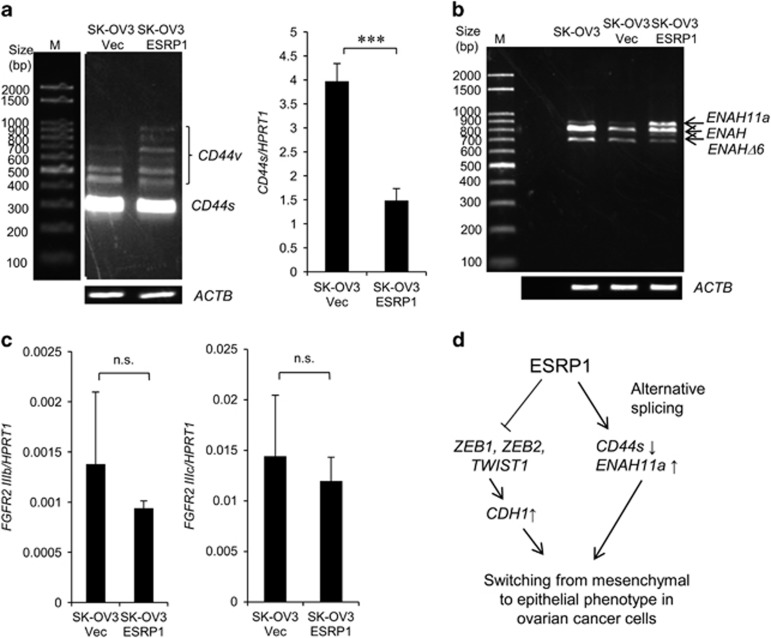
Alternative splicing of *CD44*, *ENAH* and *FGFR2* upon ectopic ESRP1 expression in mesenchymal ovarian cancer cells. (**a**) RT–PCR analysis of splice variant *CD44* (left) and qRT–PCR analysis of *CD44*s (right) in SK-OV3-Vec and SK-OV3-ESRP1 cells. (**b**) RT–PCR analysis of splice variant *ENAH* in SK-OV3-Vec and SK-OV3-ESRP1 cells. M, marker. (**c**) qRT–PCR analysis of *FGFR2IIIb* and *FGFR2IIIc* in SK-OV3-Vec and SK-OV3-ESRP1 cells. Data for qRT–PCR are presented as the mean±s.d. of four experiments. Student’s *t*-test, ***P<0.001, n.s., not significant. (**d**) Schematic diagram for a switch from mesenchymal to epithelial phenotype upon ectopic ESRP1 expression in mesenchymal ovarian cancer cells.

By regulating alternative splicing, ESRP1 promotes expression of epithelial cell-specific variants of several genes such as *CD44,*^18, 24^ *ENAH* (or *hMena*)^25^ and *FGFR2*,^15^ thereby having a role in tumor progression. Thus, we investigated whether splicing isoforms of *CD44*, *ENAH* and *FGFR2* mRNA are produced upon ectopic ESRP1 expression in OC cells. *CD44* variant isoforms (*CD44v*) were increased in SK-OV3-ESRP1 cells, whereas SK-OV3-ESRP1 cells expressed significantly reduced levels of the mesenchymal cell-specific *CD44* standard isoform (*CD44s*) than SK-OV3-Vec cells (Figure 8a). In the case of *ENAH*, three isoforms were observed, including *ENAH*, *ENAH*11a and *ENAH*Δ6. The epithelial cell-specific *ENAH* variant, *ENAH11a* isoform was upregulated in SK-OV3-ESRP1 cells, whereas mesenchymal cell-specific *ENAH*Δ6 was slightly downregulated (Figure 8b). We did not observe a significant difference in the levels of epithelial cell-specific *FGFR2IIIb* and mesenchymal cell-specific *FGFR2IIIc* between SK-OV3-Vec and SK-OV3-ESRP1 cells (Figure 8c). These findings confirmed that ESRP1 promotes production of epithelial cell-specific isoform of *ENAH* and reduces mesenchymal cell-specific isoform of *CD44*, and contributes to switching from mesenchymal to epithelial phenotype.

Taken together, these results indicate that enforced ESRP1 expression in OC cells regulates both EMT markers and alternative splicing of cancer-associated genes, thereby leading to a switch from mesenchymal to epithelial phenotype, characterized by reduced cell migration (Figure 8d).

## Discussion

Although downregulation of *ESRP1* or *ESRP2* expression during the EMT process has been well documented, their expression levels and the molecular mechanisms underlying their altered expression in human cancer have not been fully elucidated. In this study, we found that ESRP1 and ESRP2 were overexpressed in OC tissues. This is consistent with a previous study, which describes their upregulation in human oral squamous cell carcinoma in comparison with the normal epithelium,^13^ but is discordant with observations of reduced ESRP1 and ESRP2 expressions in colorectal cancer tissues.^16^ Our data provide additional evidence that the expression of these factors is cancer-type-specific.

In our TCGA data analysis, we observed frequent *ESRP1* gene amplification in OC tissues. However, we did not detect any significant positive correlation between *ESRP1* gene copy number and expression in OC cells. Importantly, our data demonstrated that DNA methylation is inversely correlated with both *ESRP1* and *ESRP2* gene expression in OC cells. In OC FFPE tissues, *ESRP2* overexpression was significantly associated with DNA hypomethylation, but no significant effect was observed for *ESRP1*. To our knowledge, this is the first study to demonstrate DNA hypomethylation as an underlying mechanism of ESRP1 or ESRP2 overexpression in OC cells. The lack of correlation between *ESRP1* expression and DNA methylation or gene copy number in OC tissues may be due to the small sample size; alternatively, it may indicate that DNA methylation or gene amplification alone is not sufficient to explain ESRP1 overexpression in OC. Instead, our data imply that several mechanisms work together to upregulate or maintain ESRP1 expression. Thus, additional studies in large samples will be required to clarify the molecular mechanism underlying ESRP1 overexpression in OC.

ESRP1 has been frequently reported as a tumor suppressor in various cancers, including colorectal^14^ and pancreatic cancer.^15^ Conversely, our TCGA data analysis revealed that high *ESRP1* expression is significantly associated with shorter 5-year OS and PFS of patients with OC. Moreover, ectopic ESRP1 expression in OC cells promoted cell proliferation. Our results suggest that ESRP1 has a cancer-promoting role in OC, similar to its metastasis-promoting role in breast cancer.^18^ However, a recent study by Lu *et al.*^17^ explored the clinical significance of *ESRP1* in various cancer types using TCGA RNA-sequencing data across 13 types of cancers, including OC, but did not detect a significant association of *ESRP1* with clinical outcome in OC. Although the exact reason for this discrepancy between our conclusions and those of Lu *et al.* remains unclear, it may result from differences in the TCGA gene expression data used for each study.

Interestingly, we found that ESRP1 suppresses migration, whereas it promotes proliferation in OC cells. Notably, this phenomenon is related to the reverse of EMT process, that is, switching from mesenchymal to epithelial phenotype, and is consistent with recent findings indicating that EMT induced by ZEB1 in lung cancer cells decreases cell proliferation but increases cell motility and invasiveness.^9^ Accumulating evidence supports the idea that there is a causal link between the EMT and downregulation of cell proliferation, and that tumors that undergo the mesenchymal–epithelial transition (MET) at a metastatic site become more proliferative.^4, 26^ Our data also showed that the reduction in cell migration induced by ESRP1 is associated with *CDH1* upregulation or *VIM* downregulation and reduced transcript levels of EMT-associated transcription factors including *ZEB1*, *ZEB2* and *TWIST1*. In particular, *ZEB2* expression was substantially reduced in ESRP1-overexpressing SK-OV3 cells, indicating that *ZEB2* has a predominant role in the regulation of *CDH1* expression. We also found that cyclin D1 expression was upregulated in ESRP1-overexpression cells (data not shown). ZEB2 represses cyclin D1 expression, thereby suppressing cell cycle progression in cells undergoing the EMT.^27^ Thus, it is reasonable to assume that downregulation of *ZEB2* by ESRP1 upregulates both *CDH1* and cyclin D1 expression, thereby promoting cell proliferation and decreasing cell migration in OC cells.

This study also demonstrated that ectopic ESRP1 expression regulates alternative splicing of *CD44* and *ENAH* in OC cells. Our findings are consistent with previous studies reporting that ESRP1 causes phenotypic switching via regulation of epithelial or mesenchymal isoforms of *CD44* or *ENAH* in mouse^18^ or human breast cancer cells^24, 25^ and human bronchial epithelial cells.^9^ Importantly, levels of *ENAH11a* isoforms are positively correlated with a high proliferation index and E-cadherin expression in human primary breast tumor tissues,^25^ in agreement with our data showing that ectopic ESRP1 expression upregulates both *CDH1* and *ENAH11a* expression. However, *FGFR2* splicing was not affected by ectopic ESRP1 expression in our study in contrast to the known role of ESRP1 as *FGFR2* splicing regulator.^1^ Our results are consistent with a previous study that stable transfection of ESRP1 in human pancreatic cancer cells did not change the expression of *FGFR2IIIb* or *IIIc.*^15^ Our results suggest that *FGFR2* isoform switching by ESRP1 occurs in a cell-type-specific manner. Taken together, our data indicate that ESRP1 drives switching from mesenchymal to epithelial phenotype by regulating alternative splicing in OC cells, similar to its function in breast cancer cells, suggesting that ESPR1 has similar roles in breast and OC.

A series of critical events including the EMT and MET contribute to cancer metastasis. Although the EMT is thought to be critical for the early transition of cells to a more invasive and metastatic phenotype, the MET is important for the late stage of metastasis, that is, tumor formation in metastatic sites.^3^ Accordingly, our *in vitro* observation that ESPR1 promotes switching from mesenchymal to epithelial phenotypes suggests that this factor contributes to formation of metastatic tumors, thus explaining the association of *ESRP1* with poor outcome in OC. Our hypothesis is supported by several previous studies. Downregulated ESRP1 and ESRP2 are re-expressed in the lymph nodes, where carcinoma cells metastasize and colonize in human oral squamous cell carcinoma.^13^ *CD44v* promotes metastasis formation in rat pancreatic cancer cells,^28^ whereas *CD44s* suppresses lung colonization of mouse metastatic cancer cells.^18^ Notably, CD44v expression including CD44v6 or soluble cleaved CD44v8-10 was associated with poor prognosis in patients with advanced epithelial OC and correlated with distant metastasis or recurrence in OC.^29, 30, 31, 32^ Therefore, it is likely that ESRP1-mediated *CD44* alternative splicing has a role in OC cells and may contribute to OC progression. Moreover, comprehensive transcriptome analysis using prostate cancer cell model of metastatic colonization revealed that metastasis-associated alternative splicing events are affected by ESRP1, suggesting that it has a role in prostate cancer metastasis.^17^ However, it remains to be determined whether ESRP1 contributes to metastatic tumor formation in OC.

In summary, this study demonstrates that ESRP1 and ESRP2 are overexpressed in OC tissues, and that DNA hypomethylation correlates with high expression of these genes in OC cells. Moreover, TCGA data analysis revealed that high *ESRP1* expression is significantly associated with poor patient outcome in OC. Enforced ESRP1 expression promoted cell proliferation, but inhibited cell migration in mesenchymal OC cells. Our data indicate that ESRP1 drives switching from mesenchymal to epithelial phenotype in OC cells via upregulation of *CDH1* expression and induction of the epithelial isoform of *ENAH*. Our findings provide the first evidence for DNA hypomethylation as the molecular mechanism underlying overexpression of ESRP1 and ESRP2, and reveal the role of ESRP1 in switching from mesenchymal to epithelial phenotypes in OC cells. These observations suggest that an epigenetic mechanism is involved in ESRP1 and ESRP2 overexpression, and that ESRP1 has a cancer-promoting role in OC.

## Materials and methods

### TCGA data analysis

A TCGA file (TCGA_OV_G4502A_07_3-2015-02-24.tgz), including normalized gene expression data based on Agilent microarrays (Agilent Technologies, Santa Clara, CA, USA) and clinical data for ovarian serous cystadenocarcinomas, was obtained from the UCSC Cancer Genome Browser (https://genome-cancer.ucsc.edu/). Of 564 samples, data from 541 primary tumor samples with clinical information were used for survival analysis. Gene expression data from four normal ovarian tissue samples were also used in this analysis. For survival analysis, the patients with ovarian serous cystadenocarcinoma were divided into two groups, *ESRP1* low expression (*ESRP1*-low) and *ESRP1* high expression (*ESRP1*-high) groups based on the cutoff values of *ESRP1* gene expression. The optimal cutoff values were determined as the point where the sum of sensitivity and specificity was maximized. A patient was assigned to the ‘*ESRP1*-high’ group when the expression value was higher than the cutoff value. Otherwise, the sample was categorized as ‘*ESRP1*-low’ group.

### Patient information and tissues

Ovarian tissue samples were obtained from patients with ovarian carcinoma following ethical approval by the Institutional Review Board of the Samsung Medical Center (Seoul, Korea). This study was performed in accordance with the Declaration of Helsinki. The study was retrospective, and informed consents from the patients involved in the study were not required as per the Institutional Review Board guidelines. Patient information was anonymized and de-identified before analysis.

### Immunohistochemistry

Immunohistochemistry for ESRP1 and ESRP2 in 93 ovarian serous carcinoma FFPE tissues was performed as described previously^33^ using anti-ESRP1 (1:100; Sigma, St Louis, MO, USA; HPA023720) and anti-ESRP2 antibodies (1:200; Abcam, Cambridge, UK; ab113486). Immunohistochemical staining was scored by a pathologist (J-SC). Cases in which the percentage of stained cells was less than 10% with weak intensity were regarded as negative (score 0). In sections in which more than 10% of the tumor cells stained, the sections were scored by the staining intensity, which was classified as follows: 1, weak; 2, moderate; and 3, strong. Cases with a score of 0 or 1 were regarded as ESRP1- or ESRP2-low, whereas cases with a score of 2 or 3 were regarded as ESRP1- or ESRP2-high. Cytoplasmic and nuclear stainings were analyzed independently.

### Cell lines and drug treatment

Human OC cell lines, IOSE cell lines and primary cultures of human normal ovarian surface epithelial cells were obtained as described previously.^34^ A2780 cells were kindly provided by Dr Stephen B Howell of University of California, San Diego (La Jolla, CA, USA). Cells were tested for *Mycoplasma* contamination. For the treatments of cells with epigenetic drugs, cells were treated with 5 μM 3-deazaneplanocin A (Dr Victor E Marquez of National Cancer Institute, MD, USA) or 5-aza-2′-deoxycytidine (Sigma) for 72 h and 200 nM trichostatin A (Sigma) for 24 h.

### Quantitative reverse transcription–PCR

Total RNA was extracted from ovarian cells using the Trizol reagent (Invitrogen, Carlsbad, CA, USA) or ovarian FFPE tissues using RecoverAll Total Nucleic Acid Isolation Kit for FFPE (Ambion, Carlsbad, CA, USA). First-strand cDNA was synthesized form RNA using superscript III reverse transcriptase (Invitrogen) or GoScript Reverse Transcription System (Promega, Madison, WI, USA), and was used for RT–PCR or qRT–PCR. qRT–PCR was carried out with FastStart Essential DNA Green Master (Roche, Mannheim, Germany) for SYBR Green in the LightCycler 96 Instrument (Roche). Primers for RT–PCR or qRT–PCR are listed in Supplementary Table 1.

### Gene copy number assay

Genomic DNA was extracted from cells using DNeasy Blood and Tissue Kit (Qiagen GmbH, Hilden, Germany), or OC FFPE tissues using the ChargeSwitch gDNA Micro Tissue Kit (Invitrogen). *ESRP1* primers for gene copy number assays were purchased from Qiagen (qBiomarker Copy number PCR assay, 337812VPH108-0478267A and 337812VPH108-0478433A). Relative quantification of gene copy number was calculated by comparing the Cq value of samples with the copy number control, albumin (*ALB*) located at 4q11-q13, followed by normalization to the calibrator. Human female genomic DNA (Promega) was used as a calibrator sample.

### Bisulfite sequencing and quantitative methylation-specific PCR

Genomic DNA extracted from OC cells or FFPE tissues was bisulfite-converted using EZ DNA Methylation-Gold Kit (Zymo Research, Orange, CA, USA) for cells and EZ DNA Methylation-Direct Kit (Zymo Research) for FFPE tissues. CpG islands in *ESRP1* and *ESRP2* promoter region and primers for bisulfite sequencing or qMSP were predicted using the Methyl Primer Express Software (Life Technologies, Carlsbad, CA, USA). Bisulfite-modified DNA was amplified by bisulfite sequencing or qMSP primers provided in Supplementary Table 2. For bisulfite sequencing, the PCR products were cloned and subsequently sequenced. DNA methylation level by qMSP was expressed as percentage of methylated reference (%) values as described previously.^34^ Methylated human control DNA from EpiTect PCR Control DNA Set (Qiagen) was used as positive control.

### Western blotting

Whole-cell lysates were extracted using RIPA buffer and used for immunoblotting according to standard procedures using primary antibody against ESRP1 (Sigma). β-actin (Santa Cruz Biotechnology, Dallas, TX, USA; sc-47778) was used as a loading control.

### DNA transfection and establishment of ESRP1-overexpressing stable cell line

Plasmid pCMV-Tag2B-ESRP1 was constructed by cloning the ESRP1-coding region to pCMV-Tag2B vector (Enzynomics, Daejeon, Korea). Cells were transfected with the plasmids using Genefectine (Genetrone Biotech, Gyeonggi-do, Korea) or Lipofectamine 2000 (Invitrogen). ESRP1-overexpressing stable clones of SK-OV3 cells were selected using Geneticin (Invitrogen) and were validated for expression using qRT–PCR and western blotting.

### siRNA transfection

The siRNA targeting *ESRP1* and non-targeting control siRNA were purchased from Dharmacon (Lafayette, CO, USA). Cells were treated with 25 nM final concentration of siRNA using Dharmafect 1 (Dharmacon) according to the manufacturer’s instruction.

### Cell proliferation and transwell migration

For cell proliferation assay, total live cells were counted using 0.4% Trypan blue staining at each time point. Migration was assessed using Costar transwell chambers with 8-mm diameter pores (Corning, NY, USA) as described previously.^35^ After 24 or 48 h, migrated cells were counted or detached from the transwell by cell detachment buffer and were quantified by CyQuant dye (Millipore, Temecula, CA, USA).

### Soft agar colony formation assay

A soft agar colony formation assay was performed using a CytoSelect 96-well Cell Transformation Assay kit (Cell Biolabs, San Diego, CA, USA) following the manufacturer's instructions. Five thousand cells per well were used and the 96-well plates were incubated for 7 days at 37 °C, 5% CO_2_. Colonies were lysed and detected with CyQuant GR dye using a fluorometer equipped with a 485/520 nm filter set (Infinite M200 Pro, TECAN, Männedorf, Switzerland). Colonies were stained with 0.05% crystal violet and photographed with a Zeiss microscope EZ 2.0 (Carl Zeiss, AG, Germany).

### Statistical analysis

Data are presented as mean±s.d. Statistical analyses comparing two groups were performed using the unpaired Student’s *t*-test or the Mann–Whitney test. Data were tested for normality and equality of variance. For survival analysis, Kaplan–Meier analysis and the log-rank test were used. OS was defined as the time from the date of diagnosis to the date of death or last follow-up, and PFS as the time from the date of diagnosis to the date of recurrence or progression. All statistical analyses were performed using R3.2.0 (http://r-project.org) or SPSS23 statistic software for Windows (SPSS, Chicago, IL, USA). *P-*values<0.05 were considered to be statistically significant. All *P*-values correspond to two-sided significance tests.
