# ESRP1 is overexpressed in ovarian cancer and promotes switching from mesenchymal to epithelial phenotype in ovarian cancer cells

**DOI:** 10.1038/oncsis.2017.89

**Published:** 2017-11-20

**Authors:** H M Jeong, J Han, S H Lee, H-J Park, H J Lee, J-S Choi, Y M Lee, Y-L Choi, Y K Shin, M J Kwon

**Correction to:** *Oncogenesis* (2017) **6**, e389; doi:10.1038/oncsis.2017.87; Published online 9 October 2017

The legend of Figure 5 was published incorrectly. The correct legend should read as follows:

**Figure 5.** Association between *ESRP1* gene expression and OS or PFS in patients with ovarian serous adenocarcinoma. (**a**, **b**) Kaplan–Meier plot of 5-year OS, and PFS in total primary tumors and (**c**, **d**) stage III primary tumors. Patients were divided into two groups based on *ESRP1* expression, *ESRP1* low expression (*ESRP1*-low) and *ESRP1* high expression (*ESRP1*-high) groups. The statistical difference in the survival between the two groups was tested with the log-rank test. The number of patients for each group is provided in parentheses.

This error has now been rectified and the corrected article appears in this issue together with this corrigendum.

The publishers wish to apologise for any inconvenience caused.

