# Correction: ESRP1 is overexpressed in ovarian cancer and promotes switching from mesenchymal to epithelial phenotype in ovarian cancer cells

**DOI:** 10.1038/s41389-019-0155-x

**Published:** 2019-08-29

**Authors:** H. M. Jeong, J. Han, S. H. Lee, H.-J. Park, H. J. Lee, J.-S. Choi, Y. M. Lee, Y.-L. Choi, Y. K. Shin, M. J. Kwon

**Affiliations:** 1 Laboratory of Molecular Pathology and Cancer Genomics, College of Pharmacy, Seoul National University, Seoul, Korea; 2 Gencurix Inc., 242 Digital-ro, Seoul, Korea; 3 Research Institute of Pharmaceutical Sciences, College of Pharmacy, Kyungpook National University, Daegu, Korea; 4 The Center for Anti-cancer Companion Diagnostics, Bio-MAX/N-Bio, Seoul National University, Seoul, Korea; 5 BK21 Plus KNU Multi-Omics based Creative Drug Research Team, Research Institute of Pharmaceutical Sciences, College of Pharmacy, Kyungpook National University, Daegu, Korea; 6 Laboratory of Cancer Genomics and Molecular Pathology, Samsung Medical Center, Sungkyunkwan University School of Medicine, Seoul, Korea; 7 Department of Pathology and Translational Genomics, Samsung Medical Center, Sungkyunkwan University School of Medicine, Seoul, Korea; 8 Department of Health Sciences and Technology, SAIHST, Sungkyunkwan University, Seoul, Korea; 9 Department of Molecular Medicine and Biopharmaceutical Sciences, Graduate School of Convergence Science and Technology, Seoul National University, Seoul, Korea; 10 College of Pharmacy, Kyungpook National University, Daegu, Korea

**Keywords:** Ovarian cancer, Oncogenes

**Correction to: Oncogenesis**

10.1038/oncsis.2017.87 Published online 9 October 2017

Since publication of the original article, the authors have noticed that there were errors in the labelling of Fig. 6d, e. The correct figure and its legend are reproduced here. The authors wish to apologise for any inconvenience caused.

**Fig. 6:**
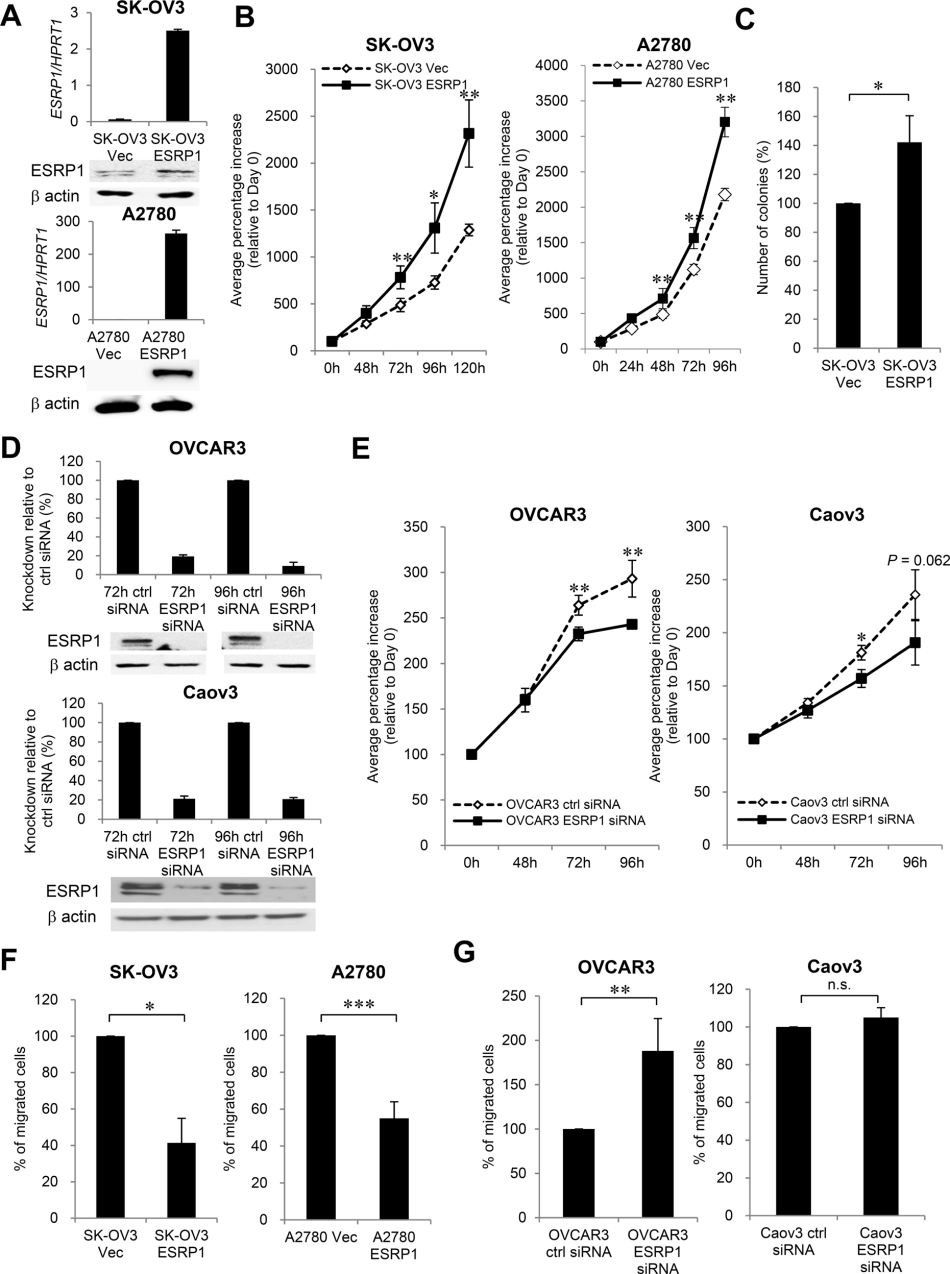
Effect of ESRP1 on cell proliferation and migration in ovarian cancer cells. **a**, **b** Effect of enforced ESRP1 expression on cell proliferation in SK-OV3 and A2780 cells. **c** Soft agar formation assay in SK-OV3 stable cell lines. **d**, **e** Effect of ESRP1 knockdown on cell proliferation in OVCAR3 and Caov3 cells. ESRP1 overexpression or ESRP1 knockdown by siRNA treatment was determined using qRT–PCR and western blot. For in vitro cell proliferation assay, viable cell numbers were counted at each time point. **f** Effect of enforced ESRP1 expression or (**g**) ESRP1 knockdown on cell migration. Data for cell proliferation and migration are presented as the mean ± s.d. of three or four experiments. Student’s *t*-test, *P < 0.05, **P < 0.01, ***P < 0.001, n.s. not significant

