# Supplementary material for: ESRP1 is overexpressed in ovarian cancer and promotes switching from mesenchymal to epithelial phenotype in ovarian cancer cells
Source: Oncogenesis. 2017 Oct 9;6(10):e389–. doi: 10.1038/oncsis.2017.87 (PMC5668885; doi:10.1038/oncsis.2017.87)
Supplement: Supplementary Tables [file oncsis201787x1.doc]

**ESRP1 is overexpressed in ovarian cancer and promotes switching from mesenchymal to epithelial phenotype in ovarian cancer cells**

Hae Min Jeong, Jinil Han, Sun Hee Lee, Hye-Jin Park, Hye Ji Lee, Jong-Sun Choi, You Mie Lee, Yoon-La Choi, Young Kee Shin, Mi Jeong Kwon*

*Corresponding Author : Mi Jeong Kwon (mjkwon94@knu.ac.kr)

**Supplementary Tables**

| Supplementary Table S1. Primers for qRT-PCR and conventional RT-PCR | | | | |  | |
| --- | --- | --- | --- | --- | --- | --- |
| Gene symbol | |  |  | Sequence (5->3) | |  |
| qRT-PCR | |  |  |  | |  |
|  | *ESRP1* | F |  | 5'-ACCAAGCCCTCCGACAGT-3' | | Taqman |
|  |  | R |  | 5'-TGCAGGATTTGCCTGACAT-3' | |  |
|  |  | UPL #44 |  | FAM-TGGGCAGC-Dark quencher | |  |
|  | *ESRP1* | F |  | 5’-ACAGAATGCGTTGAGGAAGC-3’ | | SYBR green |
|  |  | R |  | 5’-AGAGGGGCCGAGGAGAAT-3’ | |  |
|  | *ESRP2* | F |  | 5'-GCTGTTATCCTCCATCTACTCAAAG-3' | | Taqman |
|  |  | R |  | 5'-GTCCACCACATCAGCCTTGA-3' | |  |
|  |  | UPL# 3 |  | FAM-CCCAGCAG-Dark quencher | |  |
|  | *ESRP2* | F |  | 5’-AGGAGATGAGCCGAGTGCT-3’ | | SYBR green |
|  |  | R |  | 5’-GCTTGGAAGGTGGTGTAGGT-3’ | |  |
|  | *HPRT1* | F |  | 5'-CTCAACTTTAACTGGAAAGAATGTC-3' | | Taqman |
|  |  | R |  | 5'-TCCTTTTCACCAGCAAGCT-3' | |  |
|  |  | HPRT TM |  | YAK-TTGCTTTCCTTGGTCAGGCAGTATAATC–BBQ | |  |
|  | *HPRT1* | F |  | 5’-AGATGGTCAAGGTCGCAAG-3’ | | SYBR green |
|  |  | R |  | 5’-GTATTCATTATAGTCAAGGGCATATCC-3’ | |  |
|  | *CDH1* | F |  | 5'-CCCGGGACAACGTTTATTAC-3' | |  |
|  |  | R |  | 5'-GCTGGCTCAAGTCAAAGTCC-3' | |  |
|  | *VIM* | F |  | 5'-TACAGGAAGCTGCTGGAAGG-3' | |  |
|  |  | R |  | 5'-ACCAGAGGGAGTGAATCCAG-3' | |  |
|  | *ZEB1* | F |  | 5′-GGCATACACCTACTCAACTACGG -3′ | |  |
|  |  | R |  | 5′-TGGGCGGTGTAGAATCAGAGTC -3′ | |  |
|  | *ZEB2* | F |  | 5′-AAGCCCCATCAACCCATACAAG-3′ | |  |
|  |  | R |  | 5′-AAATTCCTGAGGAAGGCCCA-3′ | |  |
|  | *SNAI1* | F |  | 5′-TTCTCACTGCCATGGAATTCC-3 | |  |
|  |  | R |  | 5’-AGAGGGGCCGAGGAGAAT-3 | |  |
|  | *SNAI2* | F |  | 5′-GCCTCCAAAAGCCAAACTACA-3′ | |  |
|  |  | R |  | 5′-GAGGATCTCTGGTTGTGGTATGACA-3′ | |  |
|  | *TWIST1* | F |  | 5’-AGATGGTCAAGGTCGCAAG-3’ | |  |
|  |  | R |  | 5’-GTATTCATTATAGTCAAGGGCATATCC-3’ | |  |
|  | *CD44s* | F |  | 5'- CAGTGAAAGGAGCAGCACTT-3' | |  |
|  |  | R |  | 5'- TGGAATGTGTCTTGGTCTCTG -3' | |  |
|  | *FGFR2 IIIb* | F |  | 5'-CGTGGAAAAGAACGGCAGTAAATA-3' | |  |
|  |  | R |  | 5'-GAACTATTTATCCCCGAGTGCTTG-3' | |  |
|  | *FGFR2IIIc* | F |  | 5'-TGAGGACGCTGGGGAATATACG-3' | |  |
|  |  | R |  | 5'-TAGTCTGGGGAAGCTGTAATCTCCT- 3' | |  |
| RT-PCR | |  |  |  | |  |
| *CD44* | | F |  | 5′-GCACTTCAGGAGGTTACATC-3′ | |  |
|  | | R |  | 5′-ACTGCAATGCAAACTGCAAG-3′ | |  |
|  | *ENAH* | F |  | 5′GCTGGAATGGGAGAGAGAGCGCAGAATATC-3′ | |  |
|  |  | R |  | 5′-GTCAAGTCCTTCCGTCTGGACTCCATTGGC-3′ | |  |
|  | *ACTB* | F |  | 5′-GGCATCCTCACCCTGAAGTA-3′ | |  |
|  |  | R |  | 5′-GGGGTGTTGAAGGTCTCAAA-3′ | |  |
| F, forward primer; R, reverse primer; UPL, universal probe library | | | | |  | |

| Supplementary Table S2. Primers for gene copy number assay, bisulfite sequencing and qMSP | | | |
| --- | --- | --- | --- |
| Gene symbol | |  | Sequence (5' -> 3') |
| Gene copy number assay | | | |
|  | *ALB* |  |  |
|  | F |  | 5′-TGAAACATACGTTCCCAAAGAGTTT -3′ |
|  | R |  | 5′-CTCTCCTTCTCAGAAAGTGTGCATAT-3′ |
|  |  |  |  |
| Bisulfite sequencing | | | |
|  | *ESRP1* |  |  |
|  | F |  | 5'-GAATATAAAAAGGGTAGGTTTTTTG-3' |
|  | R |  | 5'-TTACCCCATTCCTTACATTTAA-3' |
|  | *ESRP2* |  |  |
|  | F |  | 5'-GGTAGGTAATTYGTTTGGAGAG-3' |
|  | R |  | 5'-AAAACTCCCTAAAAACCTCACC-3' |
|  |  |  |  |
| qMSP | |  |  |
|  | *ESRP1* |  |  |
|  | F |  | 5'-AGGAGTTAATGGGTCGGC-3' |
|  | R |  | 5'-CCGAAAAACACGCAATAACT-3' |
|  | *ESRP2* |  |  |
|  | F |  | 5'-GTCGTTTTTTTCGGGGTTAC-3' |
|  | R |  | 5'-TAAATCGATCTCGTCCGAAC-3' |
|  | *ACTB* |  |  |
|  | F |  | 5'-TGGTGATGGAGGAGGTTTAGTAAGT-3' |
|  | R |  | 5' AACCAATAAAACCTACTCCTCCCTTAA-3' |
| F, forward primer; R, reverse primer | | | |
